# Early-Life Compound Probiotic Intervention Programs Intestinal Barrier Maturation Through Indole-3-Lactic Acid in a Porcine Model

**DOI:** 10.3390/nu18172776

**Published:** 2026-08-25

**Authors:** Mingzhi Yang, Huan He, Jie Fu, Zhixin Yu, Wentao Li, Lixia Kai, Caihong Hu, Jie Feng, Tizhong Shan, Yizhen Wang, Mingliang Jin, Zeqing Lu

**Affiliations:** 1 College of Animal Sciences, Zhejiang University, Hangzhou 310058, China; 2 Key Laboratory of Molecular Animal Nutrition, Ministry of Education, Hangzhou 310058, China; 3 National Engineering Research Center of Green Feed and Healthy Breeding, Hangzhou 310058, China; 4 Zhejiang Key Laboratory of Nutrition and Breeding for High-Quality Animal Products, Hangzhou 310058, China

**Keywords:** compound probiotics, early-life intervention, intestinal barrier, indole-3-lactic acid, porcine model, gut microbiota

## Abstract

**Background**: The early-life gut microbiota plays a critical role in programming intestinal barrier function and immune homeostasis, with profound implications for long-term host health. However, the effects of early-life compound probiotic intervention on the maturation of intestinal barrier function and the underlying molecular mechanisms remain incompletely understood, particularly in large-animal models relevant to human physiology. **Methods**: In this preclinical study, 3627 neonatal piglets—a well-established translational model for human infant gut development—were orally administered a novel compound probiotic formulation comprising *Bifidobacterium longum* subsp. *infantis* BZ, *Lactobacillus plantarum* LZ, and *Pediococcus acidilactici* PZ during early life. A total of 1512 fecal samples collected at seven time points from birth to day 180 were analyzed by 16S rRNA sequencing, and 525 samples from three developmental windows (days 10, 25, and 70) were subjected to LC-MS-based metabolomics. The candidate metabolite indole-3-lactic acid (ILA) was further mechanistically validated in a DSS-induced colitis mouse model and in IPEC-J2 cells. **Results**: Early-life probiotic intervention significantly enhanced intestinal barrier integrity, as evidenced by improved intestinal morphology and upregulated expression of tight junction proteins—zonula occludens-1 (ZO-1), occludin (OCLN), and Claudin-1 (CLDN1)—in the jejunum (*p* < 0.05). Notably, intervention at birth was more effective than post-weaning administration, and two administrations (birth + weaning) yielded superior outcomes compared with a single administration (*p* < 0.05). Microbiome analysis revealed enhanced microbial diversity and enrichment of beneficial genera during the juvenile-to-adult transition (*p* < 0.05). Metabolomic profiling identified ILA as a signature metabolite consistently elevated by probiotic supplementation. **Conclusions**: These findings provide evidence that early-life compound probiotic intervention is associated with improved intestinal barrier maturation, with ILA identified as a key candidate metabolite that may mediate this effect, as supported by functional validation in murine and cellular models. This provides a mechanistic rationale for probiotic-based strategies to support intestinal health in human infants during critical developmental windows.

## 1. Introduction

The intestinal epithelial barrier serves as a dynamic interface between the host and the gut lumen, integrating nutritional, microbial, and immune signals to maintain tissue homeostasis [1,2]. When barrier integrity is compromised, increased intestinal permeability can contribute to a range of inflammatory and metabolic conditions, making it a clinically relevant therapeutic target [3,4]. The early postnatal period is characterized by rapid microbial assembly and functional maturation of the intestinal epithelium, and there is growing evidence that disturbances during this developmental window can have lasting health consequences—a concept encapsulated by the Developmental Origins of Health and Disease (DOHaD) framework [5,6,7]. Given that tissue sampling in human infants is severely constrained by ethical and practical considerations, physiologically relevant animal models are essential for dissecting the causal relationships between early-life microbial exposures and long-term barrier function.

The pig has become an increasingly widely used translational model for human pediatric nutrition and gastrointestinal research [6,8,9,10]. Beyond the well-recognized similarities in digestive anatomy and physiology, pigs and humans share conserved patterns of intestinal immune development and microbial succession. More importantly, the overall timeline of gut maturation—from the neonatal period through weaning and into adulthood—more closely parallels human development than that of rodents, making the pig particularly suitable for investigating the long-term programming effects of early-life interventions [1,11,12,13].

Probiotics have attracted considerable interest as a practical strategy for modulating the gut ecosystem in a beneficial manner [14,15]. Among these, lactic acid bacteria—particularly *Bifidobacterium* and *Lactobacillus* species—have been the most extensively studied, owing to their well-established safety profiles and their ability to produce a diverse array of bioactive metabolites [16,17]. Nevertheless, several important questions remain unresolved. First, the majority of studies examining probiotic efficacy in large-animal models have been limited to a single developmental stage; longitudinal investigations tracking the same cohort from early life to adulthood are surprisingly scarce [5,18]. Second, while multi-strain probiotic products are increasingly used in both research and practice, it remains unclear whether the component strains act synergistically or merely additively in influencing host physiology [19,20]. Third, and of particular relevance for translational application, the optimal timing of administration—neonatal versus post-weaning—and its impact on the durability of the intervention effects have received limited systematic attention.

Over the past decade, microbial tryptophan catabolites have emerged as key signaling molecules in host–microbiota crosstalk at the mucosal interface [21,22]. Indole derivatives that activate the aryl hydrocarbon receptor (AhR) have been shown to play a pivotal role in maintaining intestinal homeostasis. Upon ligand binding, AhR translocates to the nucleus and drives the transcription of genes encoding tight junction proteins—including zonula occludens-1 (ZO-1), occludin (OCLN), and Claudin-1 (CLDN1)—thereby reinforcing epithelial integrity and attenuating local inflammatory responses [23,24]. Among these metabolites, indole-3-lactic acid (ILA) has drawn particular attention due to its potent AhR agonist activity and its production by select *Bifidobacterium* and *Lactobacillus* strains via tryptophan transamination [25,26]. However, two critical questions have yet to be addressed: (i) whether the three-strain combination used in our formulation—*B. longum* subsp. *infantis* BZ, *L. plantarum* LZ, and *P. acidilactici* PZ—collectively enhances ILA production in the gut; and (ii) whether the ILA–AhR–tight junction axis operates in a large-animal model to mediate the long-term beneficial effects of early-life probiotic intervention on barrier function.

To address these questions, we conducted a longitudinal preclinical study in the porcine model, combining 16S rRNA sequencing (1512 fecal samples spanning seven time points from birth to adulthood) with untargeted LC-MS metabolomics (525 samples from three key developmental windows). This multi-omics approach enabled us to: (i) characterize the dynamic remodeling of gut microbial communities following early-life probiotic administration; (ii) identify signature metabolites and enriched pathways, with a particular focus on tryptophan metabolism and ILA; and (iii) mechanistically validate the role of ILA in epithelial barrier regulation using a DSS-induced colitis mouse model and IPEC-J2 cells. We hypothesized that early-life administration of the compound probiotic promotes sustained intestinal barrier maturation by enhancing ILA production and upregulating tight junction proteins. By establishing this mechanistic cascade in a translational large-animal model, we aim to provide a robust scientific foundation for the rational design of probiotic-based strategies to support intestinal health during critical developmental windows and beyond.

## 2. Participants and Methods

### 2.1. Preparation and Dilution of the Novel Compound Probiotics

The novel compound probiotics (as a lyophilized powder) were prepared as follows. *Bifidobacterium longum* subsp. *infantis* BZ, *Lactobacillus plantarum* LZ, and *Pediococcus acidilactici* PZ were cultured separately for 16 h, then mixed at equal ratios according to their colony-forming unit (CFU) counts. The mixed bacterial suspension was concentrated and freeze-dried using a lyophilizer. Subsequently, an appropriate amount of maltodextrin (Sinopharm Chemical Reagent Co., Ltd., Shanghai, China) to improve palatability and water solubility was added to produce a compound probiotic powder (10 g per sachet). The total microbial content of the compound powder was on the order of 10^11^ CFU/g.

For administration, 10 g of the compound probiotic powder was diluted with 100 mL of PBS (Wuhan Servicebio Technology Co., Ltd., Wuhan, Hubei, China) and thoroughly mixed to obtain a working bacterial suspension. The entire suspension was transferred to a feeder volumetric flask and used for oral gavage of piglets.

### 2.2. Animal Experiment Design

All animal treatments and experiments were conducted in accordance with the National Research Council’s Guide for the Care and Use of Laboratory Animals and approved by the Zhejiang University Scientific Ethics and Safety Committee (No. ZJU20220164; Hangzhou, China) on 9 May 2022.

#### 2.2.1. Porcine Preclinical Intervention Study

All animal procedures were conducted at an accredited commercial research facility (Kaihua Farm, Zhejiang Tianpeng Group Co., Ltd., China) and were approved by the Institutional Animal Care and Use Committee of Zhejiang University. A total of 3627 neonatal piglets (Large White × Landrace × Duroc) from three consecutive farrowing batches were enrolled in this longitudinal preclinical study. Piglets were randomly assigned at birth to four intervention arms (Groups A–D), with approximately 900 piglets per group at enrollment. Group A received placebo at both birth and weaning (birth−/weaning−); Group B received placebo at birth and probiotic at weaning (birth−/weaning+); Group C received probiotic at birth and placebo at weaning (birth+/weaning−); and Group D received probiotic at both time points (birth+/weaning+). The probiotic suspension (10 g lyophilized powder containing approximately 1 × 10^11^ CFU/g total bacteria, reconstituted in 100 mL PBS) was administered via oral gavage at a volume of 2 mL per piglet per administration.

Sows were moved into farrowing crates one week prior to expected parturition and were fed standard commercial lactation diets. Piglets had ad libitum access to milk from their dams and were provided with creep feed from day 10 onward. At weaning (day 26), piglets were transferred to nursery pens and gradually transitioned to a standard commercial diet regimen (ingredients and nutritional compositions are detailed in Supplementary Tables S1 and S2). All animals were housed under environmentally controlled conditions with temperature maintained within the thermoneutral zone for each developmental stage via forced-air ventilation, evaporative cooling, and supplemental heating as needed. Stocking densities and ventilation rates were in accordance with standard commercial practices for growing pigs. Animals that developed clinical illness unrelated to the experimental interventions were removed from the study cohort between stages.

Fecal samples were collected at seven time points—days 1 (via rectal swabs), 10, 25, 45, 70, 120, and 180—representing the neonatal, pre-weaning, post-weaning, juvenile, and adult stages of development. A total of 1512 fecal samples were obtained (*n* = 224, 280, 280, 96, 232, 200, and 200 at each time point, respectively) and immediately stored at −80 °C until further processing. Of these, 525 samples from days 10, 25, and 70 (*n* = 100, 240, and 185, respectively) were selected for metabolomic profiling, while all 1512 samples were subjected to 16S rRNA gene sequencing for microbiome analysis.

#### 2.2.2. Murine Model for Mechanistic Validation

Specific pathogen-free male C57BL/6J mice (4–5 weeks old, GemPharmatech, Beijing, China) were housed at the Zhejiang University Laboratory Animal Center under standard SPF conditions (12-h light/dark cycle, 22 ± 2 °C, 50 ± 10% relative humidity) with ad libitum access to a standard chow diet and water. Following a one-week acclimation period, mice were randomly assigned to four experimental groups (*n* = 8 per group, housed 4 per cage): PW (PBS gavage + sterile drinking water), PD (PBS gavage + 3% DSS in drinking water), IW (ILA gavage at 40 mg/kg body weight + sterile water), and ID (ILA gavage at 40 mg/kg + 3% DSS). During the first 14 days (pre-treatment phase), mice in the PW and PD groups received daily oral gavage of 100 μL sterile PBS, while those in the IW and ID groups received ILA (dissolved in PBS) at a dose of 40 mg/kg body weight once daily. For the subsequent 7 days (induction phase), mice in the PW and IW groups were given sterile water ad libitum, whereas the PD and ID groups received 3% DSS (*w*/*v*) in their drinking water ad libitum to induce acute colitis. The total experimental period was 21 days, after which all mice were euthanized for tissue collection.

### 2.3. DNA Extraction and Amplicon Library Construction and 16S Sequencing

The methods and steps referred to the papers of other scholars [27,28,29]. Total microb6789ial genomic DNA was extracted from fecal samples using the FastDNA^®^ SPIN Kit (MP Biomedicals Ltd., Irvine, CA, USA) according to the manufacturer’s instructions. The hypervariable region V3-V4 of the bacterial 16S rRNA gene were amplified with primer pairs 338F (5′-ACTCCTACGGGAGGCAGCAG-3′) and 806R (5′-GGACTACHVGGGTWTCTAAT-3′) [30] by an ABI GeneAmp^®^ 9700 PCR thermocycler (ABI, Redlands, CA, USA). PCR amplification cycling conditions were as follows: initial denaturation at 95 °C for 3 min, followed by 27 cycles of denaturing at 95 °C for 30 s, annealing at 55 °C for 30 s and extension at 72 °C for 45 s, and single extension at 72 °C for 10 min, and end at 4 °C. PCR products were then extracted, purified and quantified. Purified amplicons were pooled in equimolar amounts and paired-end sequenced on an Illumina MiSeq PE300 platform (Illumina, San Diego, CA, USA).

The raw reads were deposited into the NCBI Sequence Read Archive (SRA) database. Bioinformatic analysis of the fecal microbiota was carried out using the Majorbio Cloud platform (https://cloud.majorbio.com (accessed on 19 August 2023)). Quality filtering, chimera removal, and OTU clustering (at 97% similarity) were performed using UPARSE (v.7.1) with default parameters. Taxonomic assignment was performed using the SILVA database (release 138) with a confidence threshold of 0.7. Based on the OTU information, alpha diversity indices including Chao1 richness and Shannon index were calculated with Mothur v1.30.1. Beta diversity was evaluated by principal coordinate analysis (PCoA) based on unweighted UniFrac distances using the Vegan v2.5-3 package, and permutational multivariate analysis of variance (PERMANOVA) with 999 permutations was used to test for significant differences among groups. The linear discriminant analysis (LDA) effect size (LEfSe) was performed to identify the significantly abundant taxa (phylum to species) among the different groups (LDA score > 2, *p* < 0.05).

### 2.4. LC/MS Data Processing and Differential Metabolites Identification

The methods and steps were referred to the papers of other scholars [29,31,32]. The metabolites were extracted using a 400 µL methanol: water (4:1, *v*/*v*) solution with 0.02 mg/mL L-2-chlorophenylalanine as an internal standard. After protein precipitation and centrifugation, the supernatant was carefully transferred to sample vials for LC-MS/MS analysis. A pooled quality control sample (QC) was prepared by mixing equal volumes of all samples. The QC samples were disposed and tested in the same manner as the analytic samples. The instrument platform for this LC-MS analysis is a UHPLC-Q Exactive HF-X system of Thermo Fisher Scientific (Waltham, MA, USA). After the mass spectrometry detection is completed, the raw data of LC/MS is preprocessed by Progenesis QI (Version 3.0, Waters Corporation, Milford, MA, USA) software. At the same time, the metabolites were searched, identified and analyzed.

### 2.5. Measurement of Intestinal Health-Related Indicators

#### 2.5.1. H&E Staining

Fresh jejunum and colon tissues were taken and fixed with 4% paraformaldehyde. The tissues were then removed and placed flat in a dehydration box. The jejunum and colon tissues in the dehydration box were washed with PBS, then dehydrated in ethanol with different concentration gradients, eluted with xylene three times, about 10 min each time, and then eluted three times with paraffin, each time 1 h. An embedding machine was used to embed and trim tissue samples. The slice thickness was adjusted to 4 μm, placed flat on a glass slide, and then dried in an oven at 60 °C. Stained with hematoxylin for 15 min, followed by staining with eosin for 1 min, dehydrated and sealed; then the jejunum and colon sections were observed under different microscope magnifications and pictures.

#### 2.5.2. Measurement of Tight Junction Proteins and Their Gene Expression

Quantitative real-time PCR was used to detect the expression of tight junction protein genes. Detailed steps refer to our previous research [33]. For quantitative real-time PCR, the general steps were RNA extraction, reverse transcription into cDNA, experiment on the instrument and calculating the relative expression of the target gene. Probiotics colonization detection also used quantitative real-time PCR methods. All primer information was shown in Supplementary Table S3.

#### 2.5.3. Measurement of Short-Chain Fatty Acids in Pig Feces

Treatment method: weighed the pig feces sample, added 500 µL methanol solution into the EP tube, stood for 10 min, shook and mixed to make a feces suspension, adjusted the pH to 2–3 with sulfuric acid, stood for 5 min, and repeated several times, centrifuge the EP tube at 4 °C and 5000 rpm for 20 min, took the supernatant and centrifuge again for 5 min, and used GC-MS for detection.

Instrument conditions: carrier gas was helium, flow rate was 1 mL/min, initial column temperature was 100 °C, and maintained for 0.5 min. Then it was raised to 180 °C at a rate of 8 °C/min and maintained for 1 min. Then increase to 200 °C at a speed of 20 °C/min and maintained for 1 min. The detector temperature was controlled at 240 °C, the inlet was set at 200 °C, and the injection volume was 1 µL.

#### 2.5.4. Cell Culture Experiment

IPEC-J2 cells were first treated with ILA at concentrations of 0.2 and 0.5 mM to assess cell viability. Based on these results, 0.5 mM was selected as the optimal concentration for subsequent evaluation of the mRNA expression of ZO-1, Occludin, and Claudin-1.

#### 2.5.5. Probiotic Strain Quantification by qPCR

For quantification of the three probiotic strains in fecal samples, strain-specific primers targeting the 16S rRNA genes of *B. longum* subsp. *infantis* BZ, *L. plantarum* LZ, and *P. acidilactici* PZ were designed (Supplementary Table S3). Total bacterial DNA was extracted as described above. qPCR was performed using a LightCycler^®^ 480 System (Roche, Basel, Switzerland) with the following cycling conditions: 95 °C for 5 min, followed by 40 cycles of 95 °C for 15 s, 60 °C for 30 s, and 72 °C for 30 s. Standard curves were generated using serial 10-fold dilutions of purified PCR products (10^2^–10^8^ copies/µL). The copy number of each target gene per µL of extracted DNA was calculated using the standard curve method. All samples were analyzed in triplicate, and results are expressed as log_10_(copies/µL DNA).

### 2.6. Calculation and Statistical Analysis

Spearman correlation analysis was generated by calculating the Spearman correlation coefficient between microbial composition (phylum and genus level). SPSS 23.0 software was used for statistical analysis of data, *t*-test analysis was performed between two groups, one-way ANOVA analysis was performed between multiple groups, and multiple comparisons were performed by Duncan’s method. All data were expressed as mean ± standard error. The superscripts without the same lowercase letters indicated significant differences (*p* < 0.05). ns meant there was no significant difference between the groups. The data were then plotted using Graphpad Prism 7.0.

## 3. Results

### 3.1. Probiotic Intervention Improves Intestinal Morphology and Barrier Integrity

To evaluate the sustained physiological effects of early-life probiotic administration, we analyzed growth trajectories and clinical parameters from birth to adulthood (Supplementary Table S4). During the post-weaning period (day 26 to day 70), piglets in the birth-only (group B) and dual-intervention (group D) groups exhibited significantly lower diarrhea incidence than the placebo group (*p* < 0.05). By day 70, body weight in group D was significantly higher than in all other groups *(p* < 0.05). Overall, the dual-intervention regimen (group D) outperformed single administration, and birth administration (group C) was more effective than post-weaning administration (group B) in promoting long-term growth (*p* < 0.05).

**Figure 1:**
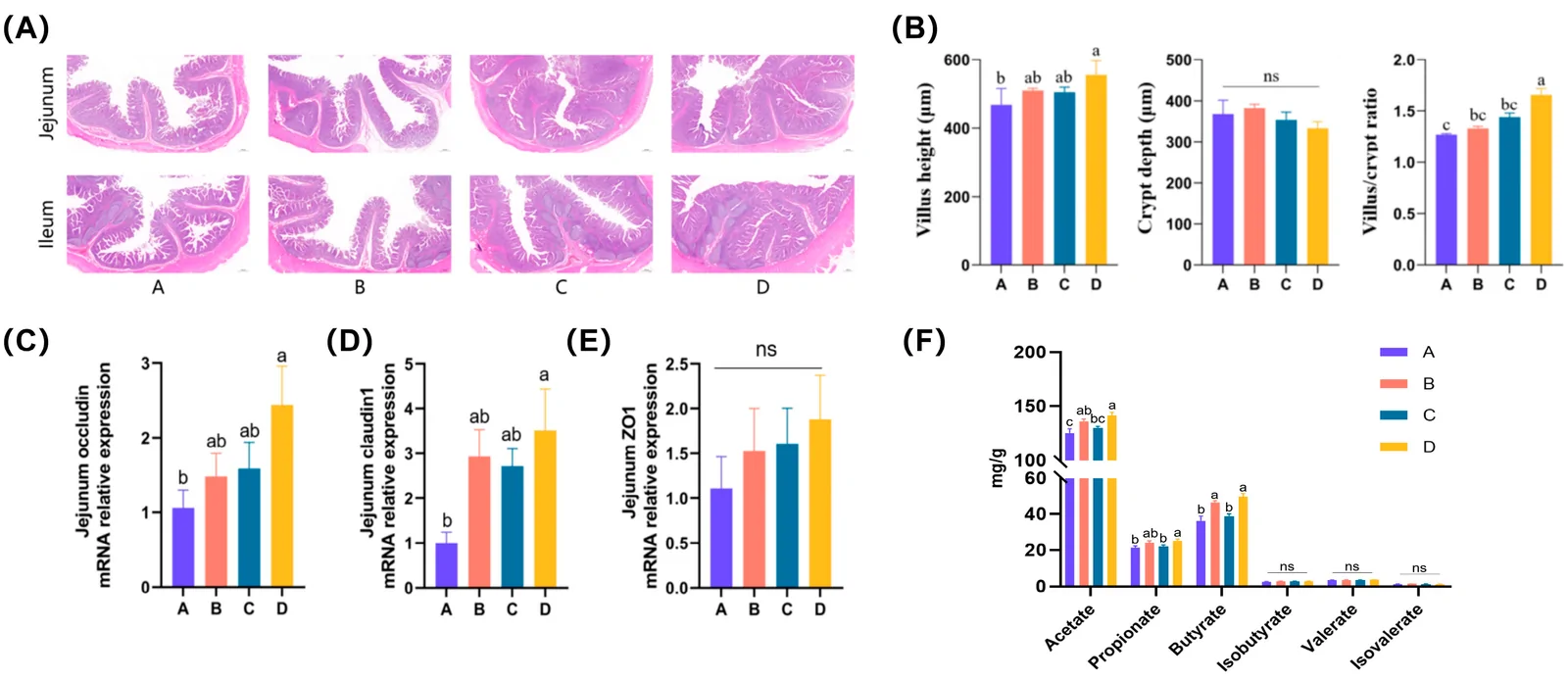
**Effects of novel compound probiotics on pig intestinal health (*n* = 6 per group).** (**A**) Jejunum and ileum morphology of experimental pigs in each group. (**B**) Jejunal villus height and crypt depth. (**C**) mRNA expression levels of Occludin as measured by qRT-PCR. (**D**) mRNA expression levels of Claudin-1 as measured by qRT-PCR. (**E**) mRNA expression levels of ZO-1 as measured by qRT-PCR. (**F**) The content of short-chain fatty acids in the feces of experimental pigs. No identical lowercase letters indicated significant differences (*p* < 0.05), and ns indicated that the differences between groups were not significant (*p* > 0.05).

We next examined whether the improved growth outcomes were accompanied by enhanced intestinal barrier function. As shown in Figure 1A, jejunal and ileal villi in groups C and D appeared more organized and densely arranged compared with groups A and B, with more distinct crypt architecture. Morphometric analysis (Figure 1B) revealed that jejunal villus height in group D was significantly greater than in group A (*p* < 0.05), with a gradual increasing trend from group A to group D. Crypt depth did not differ significantly among the four groups (*p* > 0.05), although a decreasing trend was observed from group A to group D. The villus-to-crypt ratio in group D was significantly higher than in group A (*p* < 0.05).

Quantitative PCR (Figure 1C–E) revealed that mRNA levels of *Occludin* and *Claudin-1* in group D were significantly higher than in group A (*p* < 0.05), whereas no significant differences were detected among groups A, B, and C (*p* > 0.05). No significant difference in ZO-1 mRNA expression was observed among the four groups (*p* > 0.05).

Fecal short-chain fatty acid (SCFA) profiles (Figure 1F) indicated that group D had significantly higher acetate and butyrate levels than groups A and C (*p* < 0.05), and significantly higher propionate than group A (*p* < 0.05). Overall, group D exhibited the highest levels of acetate, propionate, and butyrate among all groups. No significant differences were observed among the four groups in isopropionate, valerate, or isovalerate (*p* > 0.05).

Collectively, these results provide evidence that the compound probiotic improves intestinal morphology, increases villus height and the villus-to-crypt ratio, upregulates tight junction protein expression, and enhances SCFA production in the jejunum.

### 3.2. Probiotic Intervention Modulates Gut Microbial Diversity and Composition over Time

**Figure 2:**
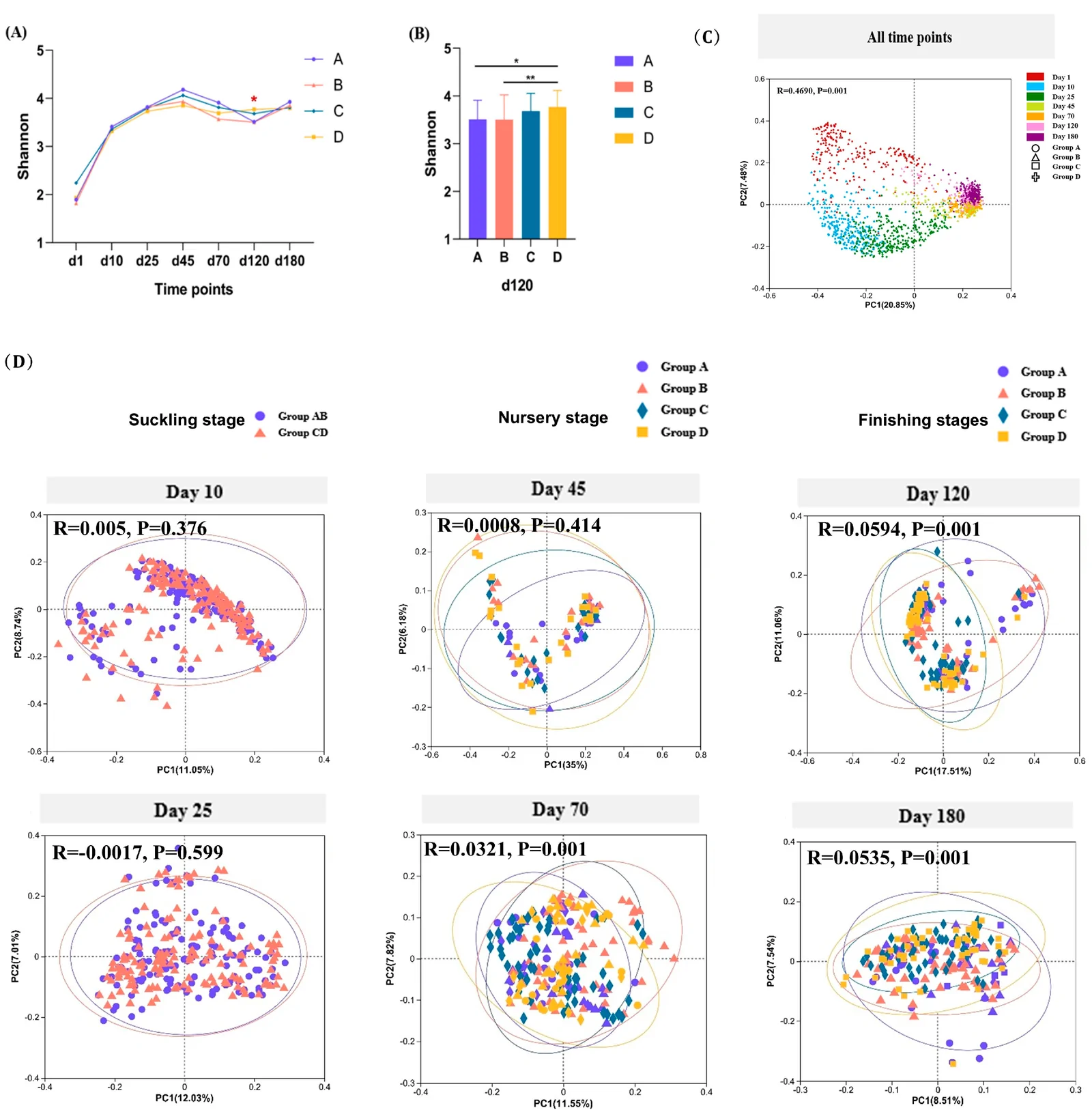
**Effects of novel compound probiotics on α-diversity and β-diversity of swine intestinal microbes.** A total of 1512 fecal samples were obtained (*n* = 224, 280, 280, 96, 232, 200, and 200 at each time point, respectively. (**A**) The Shannon index of the four groups at each time point; (**B**) the Shannon index of the four groups on day 120. (**C**) Differences in intestinal microbial composition and structure among the four groups at different time points. Different colors represented different time points, and different shapes represented different groups. The closer the distance between different samples, the more similar the microbial composition and structure were. (**D**) Differences in intestinal microbial composition and structure between groups at different stages. Statistical significance was considered at *p* < 0.05, with the following significance levels: * *p* < 0.05, ** *p* < 0.01.

The effects of the compound probiotic on intestinal microbial α-diversity (Shannon index) across different time points are presented in Figure 2A. The Shannon index increased markedly from day 1 through day 45 across all groups. Among the four groups, significant differences were observed only on day 120 (Figure 2B), where group D exhibited a significantly higher Shannon index than groups A and B (*p* < 0.05). Although group A showed numerically higher Shannon indices than groups B, C, and D on days 70 and 180, these differences did not reach statistical significance (*p* > 0.05). These results suggest that the probiotic intervention enhanced microbial diversity during the later developmental stages.

β-diversity analysis (Figure 2C,D) revealed that temporal changes in microbial community structure were more pronounced than inter-group differences, indicating that developmental stage exerted a greater influence on gut microbial composition than probiotic treatment. Significant shifts in microbial composition were observed among days 1, 10, 25, and 45, whereas no significant differences were detected among days 45, 70, 120, and 180, suggesting that the gut microbial community stabilized from approximately day 45 onward (Figure 2C).

When comparing groups at each time point (days 10 and 25 for the neonatal period; days 45 and 70 for the post-weaning period; days 120 and 180 for the juvenile-to-adult period), no significant differences were observed among groups on days 10, 25, or 45 (*p* > 0.05). In contrast, significant inter-group differences emerged on days 70, 120, and 180 (*p* < 0.05, Figure 2D), indicating that the probiotic intervention gradually reshaped the gut microbial composition during the later developmental stages.

### 3.3. Probiotic Intervention Alters Gut Microbial Composition at Phylum and Genus Levels

**Figure 3:**
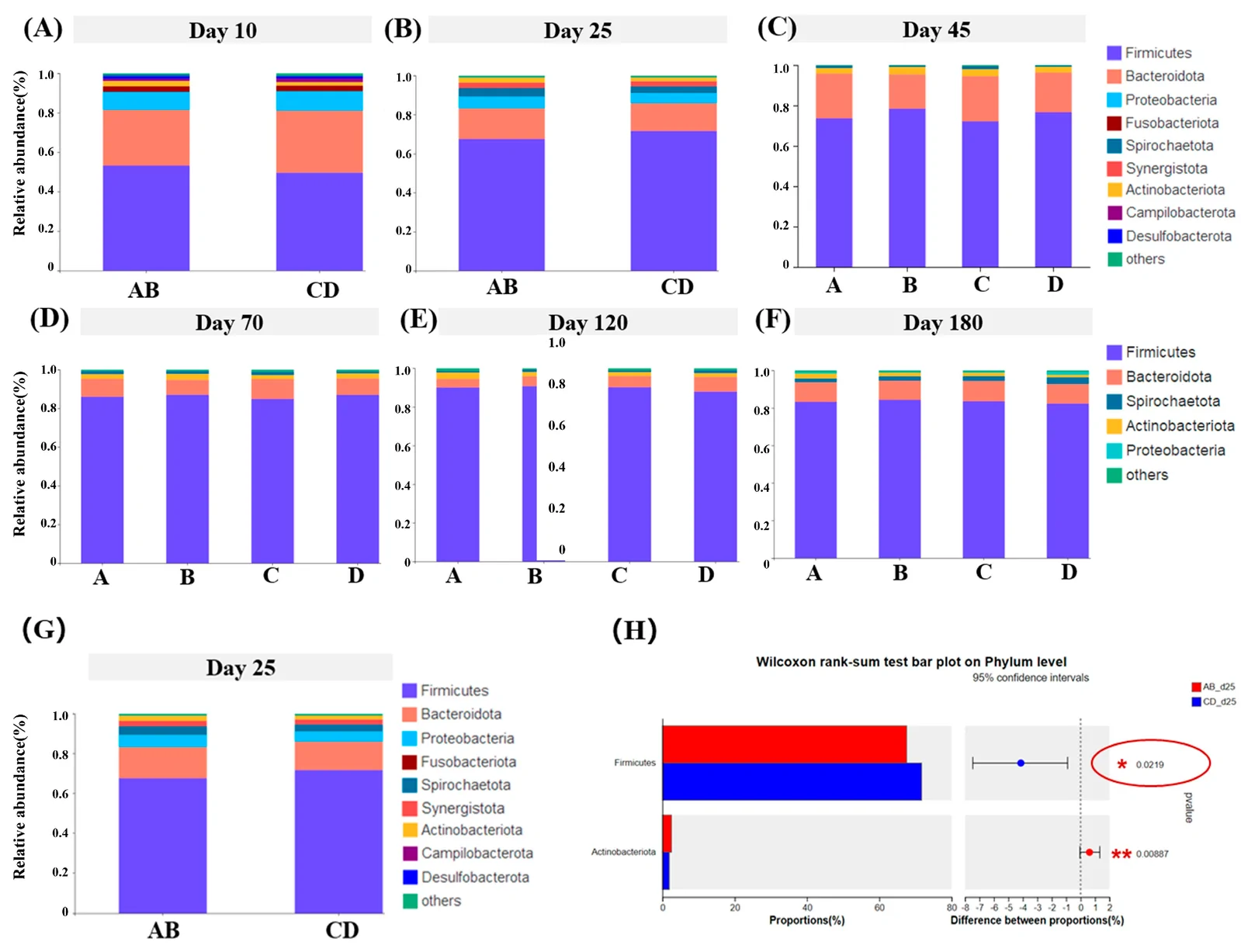
**Effects of novel compound probiotics on the phylum level of porcine intestinal microbes at different time points.** A total of 1512 fecal samples were obtained (*n* = 224, 280, 280, 96, 232, 200, and 200 at each time point, respectively. (**A**–**F**) Differences at the phylum level at different time points among different groups; (**G**,**H**) Differences and significance of phylum levels between group AB and group CD on day 25. Statistical significance was considered at *p* < 0.05, with the following significance levels: * *p* < 0.05, ** *p* < 0.01.

The effects of the compound probiotic on gut microbiota at the phylum level are shown in Figure 3. From day 10 to day 180 (Figure 3A–F), Firmicutes was the predominant phylum, with relative abundance ranging from 50% to 90%, followed by Bacteroidetes (5–20%). On day 10, Firmicutes abundance in group CD was slightly lower than in group AB, but the difference was not significant (*p* > 0.05). In contrast, on day 25, Firmicutes abundance in group CD was significantly higher than in group AB (*p* < 0.05; Figure 3G,H), suggesting that the probiotic intervention enhanced Firmicutes colonization by the end of the neonatal period.

To identify differentially abundant bacterial taxa among groups, LEfSe analysis was performed on fecal samples collected at key time points (Supplementary Figure S1). On day 10, *Bifidobacterium longum* and *Pediococcus acidilactici* were significantly enriched in group CD. On day 25, *Lactobacillus coleohominis* was significantly enriched in group CD. On day 70, *Butyricicoccaceae* (family level) and *Lactobacillus mucosae* were significantly enriched in group B, while *Clostridium butyricum* and *Romboutsia* were significantly enriched in group D. On day 180, *Faecalibacterium prausnitzii* and *Coprococcus* were significantly enriched in group B, while *Peptococcus* was significantly enriched in group C.

### 3.4. Colonization Dynamics of the Three Probiotic Strains in the Gut

**Figure 4:**
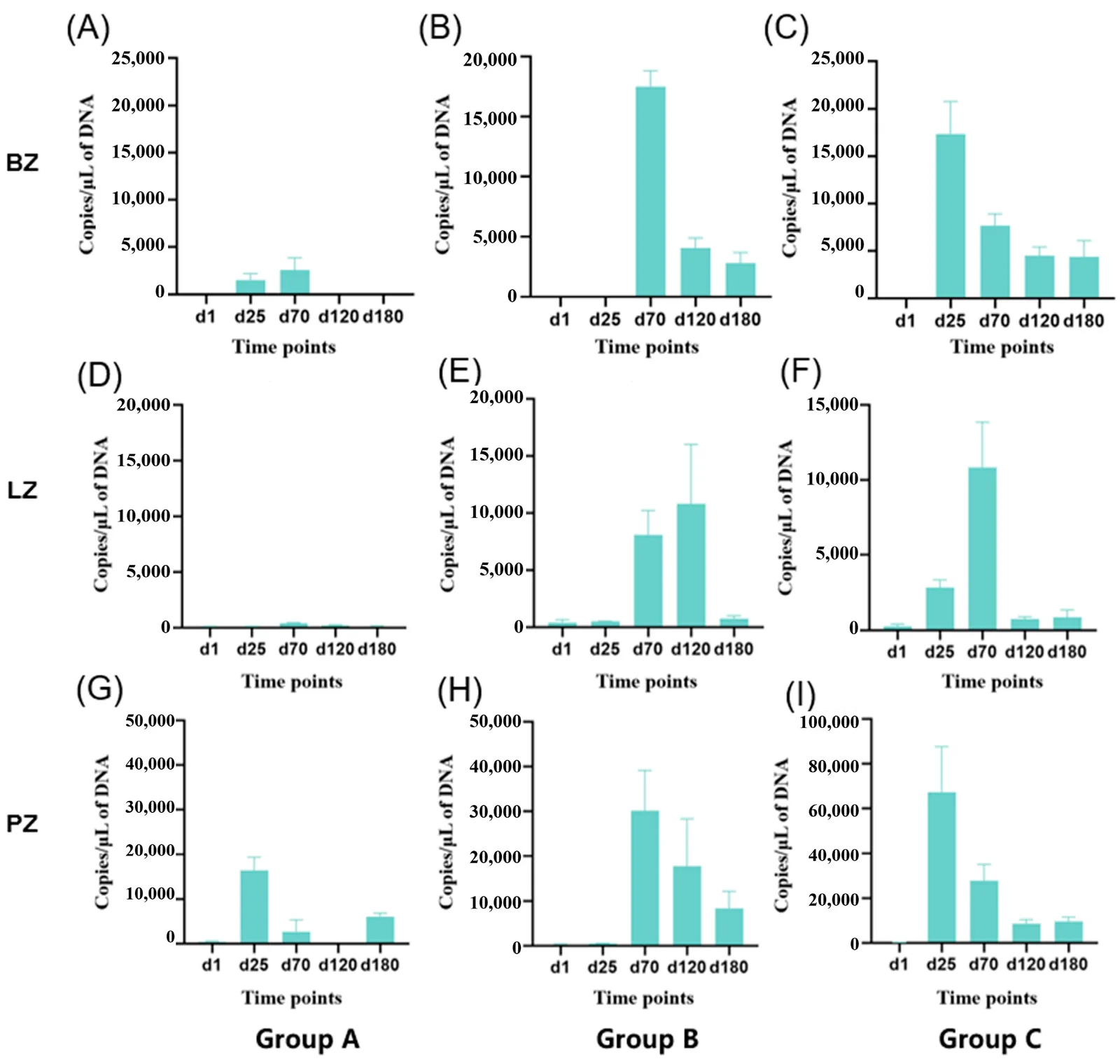
**Colonization detection of novel compound probiotics in porcine intestine (*n* = 4 at each time point).** (**A**–**C**) Colonization detection of *Bifidobacterium* BZ at each time point; (**D**–**F**) Colonization detection of *Lactobacillus plantarum* LZ at each time point; (**G**–**I**) Colonization detection of *Pediococcus acidilactici* PZ at each time point. Data are expressed as copies/µL of extracted DNA, with total DNA extracted from 200 mg of feces and eluted in 100 µL of TE buffer. Bars represent the mean ± SD.

The persistence of the three probiotic strains in the intestine was assessed by qPCR at multiple time points (Figure 4). Figure 4A–C show the colonization of *Bifidobacterium* in groups A, B, and C, respectively; Figure 4D–F show that of *Lactobacillus plantarum*; and Figure 4G–I show that of *Pediococcus acidilactici*.

When piglets received the probiotic on day 29 (3 days post-weaning, group B), all three strains were detectable in feces on days 70, 120, and 180 (Figure 4B,E,H), indicating persistence of strain-specific DNA in the gut. While this does not definitively demonstrate viability or metabolic activity, the sustained detection of all three strains through day 180 suggests long-term residence of the administered bacteria. The copy numbers of *Bifidobacterium* and *P. acidilactici* peaked on day 70 and then declined but remained detectable through day 180. In contrast, *L. plantarum* copy numbers were highest on day 120, intermediate on day 70, and still detectable on day 180.

When piglets received the probiotic at birth (group C), all three strains were detectable on days 25, 70, 120, and 180 (Figure 4C,F,I), also indicating persistence of strain-specific DNA in the gut. The copy numbers of *Bifidobacterium* and *P. acidilactici* peaked on day 25 and then declined but persisted through day 180. *L. plantarum* copy numbers were highest on day 70, followed by day 25, and remained detectable on days 120 and 180.

Collectively, these results provide evidence that all three probiotic strains—whether administered at birth (group C) or after weaning (group B)—successfully colonized the gut and persisted until day 180.

### 3.5. Metabolomic Profiling Identifies ILA as a Key Probiotic-Responsive Metabolite

**Figure 5:**
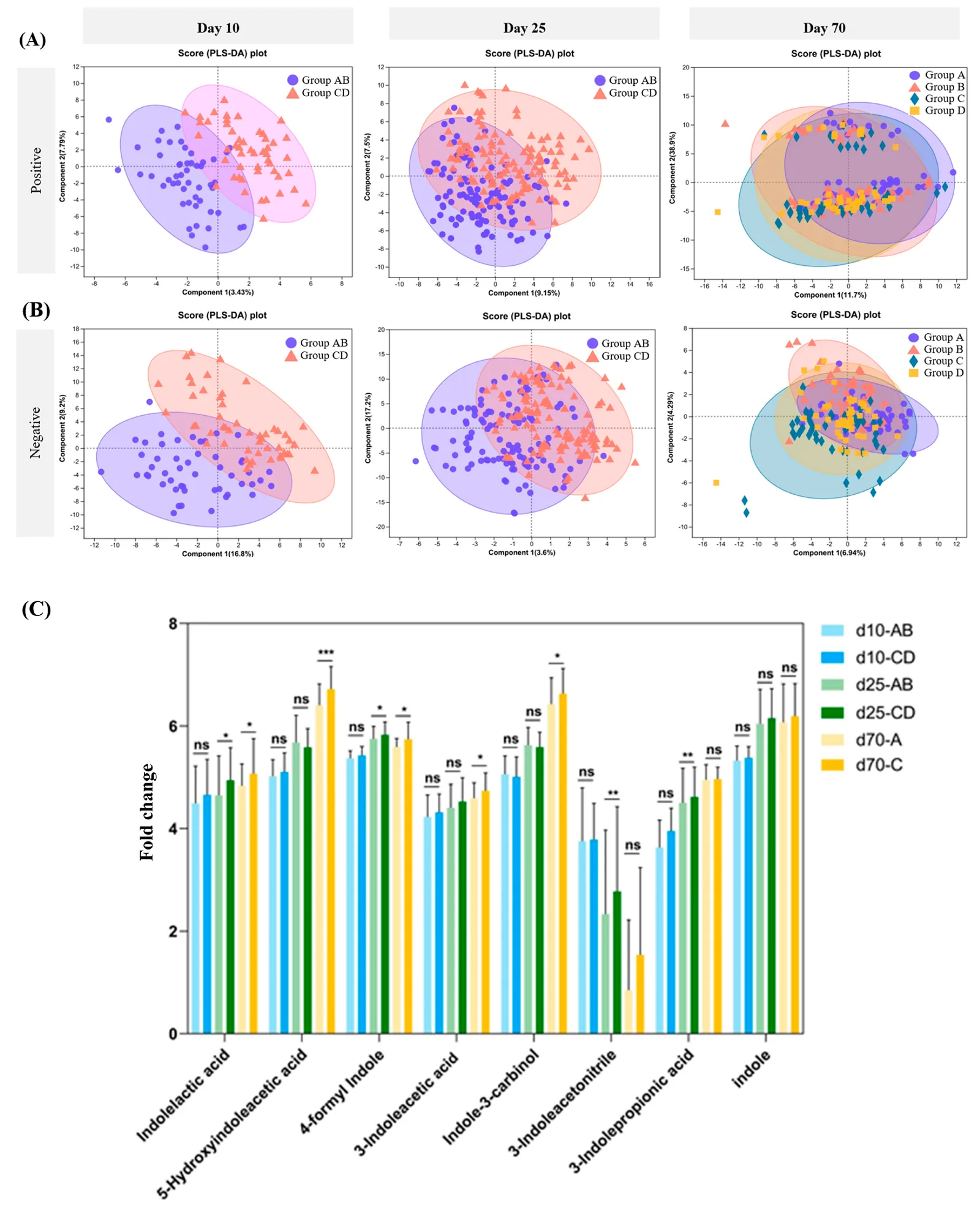
**Multivariate analysis of metabolomic profiles and probiotic-responsive indole metabolites.** 525 samples from days 10, 25, and 70 (*n* = 100, 240, and 185, respectively) were selected for metabolomic profiling. (**A**) In the positive ion mode. (**B**) In the negative ion mode. Each point represents an individual sample; the closer the points, the more similar their metabolic profiles. The x-axis indicates the first principal component (PC1), and the y-axis indicates the second principal component (PC2), with the percentage of variance explained shown in parentheses. (**C**) Fold changes in tryptophan-derived indole metabolites in the probiotic-treated groups (CD) relative to the control groups (AB) at days 10, 25, and 70, based on normalized LC-MS peak areas. Data are expressed as fold change (CD/AB). d10-AB represents the average of Groups A and B at day 10; d10-CD represents the average of Groups C and D at day 10; analogous definitions apply for d25-AB, d25-CD, d70-A, and d70-C. Statistical significance was considered at *p* < 0.05, with the following significance levels: * *p* < 0.05, ** *p* < 0.01, *** *p* < 0.001, and “ns” indicating no significant difference.

LC-MS-based metabolomic profiling was performed on fecal samples collected on days 10, 25, and 70. PLS-DA analysis clearly distinguished the metabolite profiles among the four groups in both positive and negative ion modes at each time point (Figure 5A,B).

On day 10, metabolites such as LysoPE and 2-isopropylmalic acid were significantly increased in group CD, whereas 2-[3-(sulfooxy)phenyl]acetic acid and Corey PG-lactone diol were significantly decreased (*p* < 0.05; Supplementary Figure S2A). Enriched pathways for these differential metabolites were primarily purine metabolism and the biosynthesis of valine, leucine, and isoleucine (Supplementary Figure S2B).

On day 25, metabolites including N1-acetylspermine and 20-HDoHE were significantly increased in group CD, while 1-cinnamoylpiperidine, ethyladipic acid, and floionolic acid were significantly decreased (*p* < 0.05; Supplementary Figure S3A). Enriched pathways included tryptophan metabolism, arginine and proline metabolism, Th1/Th2 cell differentiation, calcium signaling, and fat digestion and absorption (Supplementary Figure S3B).

On day 70, hexadecanedioic acid mono-L-carnitine ester was significantly increased in group C, whereas 3-hydroxytridecanoic acid, ethyl 3-hydroxytridecanoate, panaquinquecol 1, and estrone were significantly decreased (*p* < 0.05; Supplementary Figure S4). Enriched pathways included protein digestion and absorption, tryptophan metabolism, and the mTOR signaling pathway.

We next examined the changes in tryptophan-derived indole metabolites in groups A and C on days 10, 25, and 70 (Figure 5C). Indole-3-lactic acid (ILA) was significantly higher in group C than in group A on days 25 and 70 (*p* < 0.05), with a non-significant upward trend on day 10 (*p* > 0.05). 5-Hydroxyindoleacetic acid was significantly increased in group C only on day 70 (*p* < 0.05). 4-Formylindole was significantly higher in group C on days 25 and 70 (*p* < 0.05), with a non-significant upward trend on day 10. Indole-3-acetic acid and indole-3-carbinol were significantly higher in group C on day 70 (*p* < 0.05), while no significant differences were observed on days 10 or 25, although upward trends were noted. No significant differences were detected for 3-indoleacetonitrile or indole at any time point (*p* > 0.05), though upward trends were observed in group C. 3-Indolepropionic acid was significantly higher in group C on day 25 (*p* < 0.05), but not on days 10 or 70.

Collectively, tryptophan metabolism was consistently enriched among the differential pathways on days 25 and 70, and ILA was the most consistently elevated indole metabolite in group C. This points to ILA as a candidate metabolite through which the probiotic may influence intestinal barrier function.

### 3.6. ILA Protects Against DSS-Induced Intestinal Barrier Injury In Vivo and Upregulates Tight Junction Proteins in IPEC-J2 Cells

To test whether ILA causally mediates the observed barrier-protective effects, we next evaluated its direct action in a DSS-induced colitis mouse model and in IPEC-J2 cells. Colitis was induced by administering 3% DSS in drinking water for 7 consecutive days to groups PD and ID. Mice in groups IW and ID received daily ILA gavage (40 mg/kg) for 14 days prior to DSS treatment.

**Figure 6:**
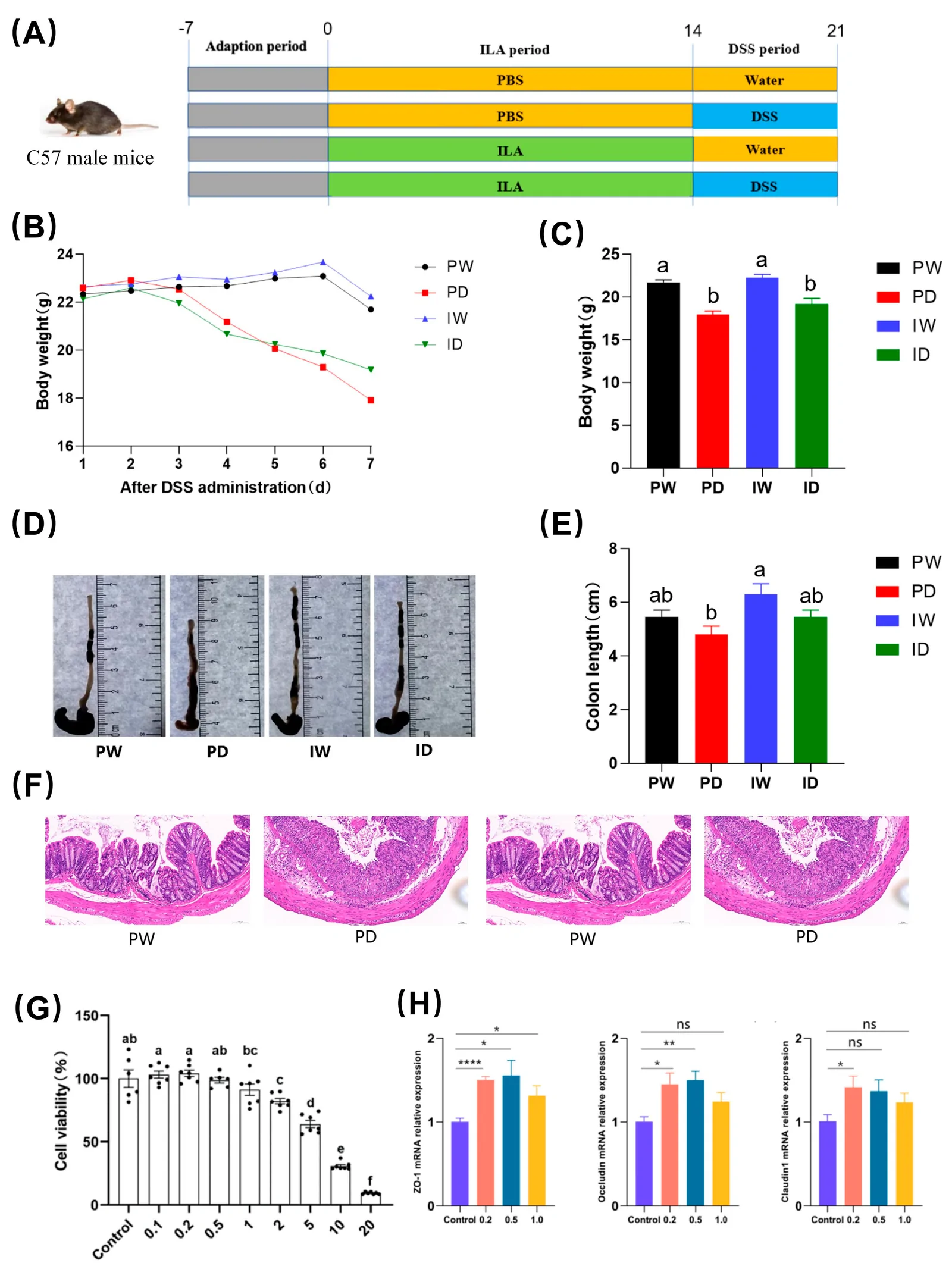
**Effects of ILA on DSS-induced colitis in mice and on tight junction protein expression in IPEC-J2 cells.** (**A**) Experimental design and treatment schedule in mice (*n* = 6 per group). (**B**) Dynamic changes in body weight during the DSS administration period. (**C**) Final body weight at the end of the experiment. (**D**,**E**) Representative images and quantitative analysis of colon length. (**F**) Representative photomicrographs of H&E-stained colon sections (150× magnification) showing histopathological changes. (**G**) Viability of IPEC-J2 cells treated with graded concentrations of ILA. (**H**) Relative mRNA expression levels of tight junction proteins (e.g., occludin, claudin-1, ZO-1) in IPEC-J2 cells after ILA treatment at different concentrations. Statistical significance was considered at *p* < 0.05, with the following significance levels: * *p* < 0.05, ** *p* < 0.01, **** *p* < 0.0001, and “ns” indicating no significant difference. No identical lowercase letters indicated significant differences (p < 0.05).

As shown in Figure 6B, body weight in the DSS-treated groups (PD and ID) decreased sharply from day 3 onward compared with non-DSS controls (PW and IW). From day 5 within the DSS-treated groups, body weight in group ID was higher than in group PD (*p* > 0.05), suggesting that ILA partially alleviated DSS-induced weight loss. At the end of the experiment (Figure 6C), DSS-treated mice had significantly lower body weight than non-DSS controls (*p* < 0.05), confirming successful colitis induction. Within the DSS-treated groups, body weight in group ID remained higher than in group PD (*p* > 0.05). Colon length measurements (Figure 6D,E) showed no significant differences among groups, although group ID exhibited a numerically longer colon than group PD.

Histological analysis (Figure 6F) revealed that group PD had severely damaged intestinal villi and disorganized colonic crypts, whereas group ID displayed more intact morphology in both the colon and jejunum. Among the non-DSS groups, group IW showed improved villus architecture compared with group PW, indicating that ILA alone enhanced intestinal morphology.

To further investigate the direct mechanism by which ILA affects intestinal epithelial cells, we conducted in vitro experiments using IPEC-J2 cells. As shown in Figure 6G, ILA at 0.1–1.0 mM did not affect cell viability (*p* > 0.05), whereas concentrations ≥2.0 mM were cytotoxic (*p* < 0.05). Based on these results, concentrations of 0.2, 0.5, and 1.0 mM were selected for subsequent experiments. At the mRNA level (Figure 6H), 0.2 and 0.5 mM ILA significantly upregulated *ZO-1* and *Occludin* (*p* < 0.05), and 0.2 mM ILA also significantly increased *Claudin-1* (*p* < 0.05).

Collectively, these results provide evidence that ILA directly acts on intestinal epithelial cells to upregulate tight junction proteins, supporting its role as a key mediator of the probiotic effects on intestinal barrier function.

## 4. Discussion

The present study provides, to our knowledge, the first comprehensive longitudinal investigation of early-life compound probiotic intervention in a translational porcine model, tracking the same cohort from birth through to adulthood. By integrating 16S rRNA sequencing, untargeted metabolomics, and mechanistic validation in both murine and cellular models, we provide evidence that a compound probiotic formulation comprising *Bifidobacterium longum* subsp. *infantis* BZ, *Lactobacillus plantarum* LZ, and *Pediococcus acidilactici* PZ exerts sustained beneficial effects on intestinal barrier function and systemic growth, with ILA identified as a key candidate metabolite that may mediate these effects. Importantly, while the three-strain mixture was administered as a single formulation, the relative contribution of each strain to the production of ILA and other bioactive metabolites remains to be systematically dissected in future studies. Beyond confirming the probiotic’s efficacy, our findings reveal two conceptually important insights: (i) the timing of administration critically determines the magnitude and persistence of the intervention outcomes; (ii) the beneficial effects are associated with sustained remodeling of tryptophan metabolism and ILA production, which directly reinforces epithelial barrier integrity as demonstrated in murine and cellular models. Collectively, these observations support a correlative and functionally validated framework for understanding how early-life microbial interventions may influence long-term intestinal homeostasis, while highlighting ILA as a central candidate in this process. Definitive proof of causality in the porcine model—e.g., through AhR blockade or ILA depletion—remains to be established in future studies.

### 4.1. The Porcine Model: A Translational Bridge for Human Infant Nutrition

A central premise of this study is the use of the pig as a translational model for human infant gut development. The suckling piglet has been extensively validated as a suitable animal model for human infants, with a digestive system, immune development, and temporal trajectory of gut maturation that closely parallel those of humans [34,35,36]. Unlike rodents, whose intestinal development occurs predominantly postnatally, pigs—like humans—undergo significant intestinal maturation during the perinatal period, making them uniquely suited for investigating the long-term programming effects of early-life nutritional interventions [37,38,39]. This developmental congruence is particularly relevant to the DOHaD paradigm, which posits that environmental exposures during critical developmental windows can program susceptibility to later-life disease [6,40,41]. By demonstrating that a single neonatal probiotic administration exerts effects that persist until adulthood (day 180), our study provides direct experimental evidence in a physiologically relevant large-animal model that early-life microbial intervention can produce lasting programming effects on host physiology—a finding with direct translational implications for human infant nutrition.

### 4.2. Early-Life Intervention Timing: Implications for the “Window of Opportunity”

One of the most striking findings of this study is the differential efficacy of probiotic administration timing: neonatal intervention (group C) was superior to post-weaning intervention (group B), and dual intervention (group D) yielded the most robust outcomes. This observation carries both biological and translational significance.

From a biological perspective, the neonatal period represents a unique window during which the gut microbiota is actively assembling and the intestinal barrier is undergoing functional maturation. The intestinal epithelium of the newborn is characterized by heightened permeability to macromolecules, immature immune responses, and a developing mucus layer—features that render this period particularly amenable to microbial modulation [42,43,44]. Our observation that birth administration promoted more persistent colonization of all three probiotic strains than post-weaning administration (Figure 4) supports the notion that early colonization exploits a “window of opportunity” during which the gut ecosystem is less competitive and more receptive to exogenous microbial input. This is consistent with the well-documented phenomenon that species administered early in life achieve more stable engraftment than when introduced later [45,46,47,48].

From a translational perspective, these findings align with emerging evidence from human studies. *B. infantis* treatment has been shown to promote weight gain in malnouritan infants, and early *Bifidobacterium* supplementation has been associated with reduced intestinal inflammation and improved immune development [49]. Our data extend these observations by demonstrating, in a controlled large-animal setting, that the *timing* of intervention—not merely the fact of supplementation—is a critical determinant of long-term efficacy. This has direct implications for the design of probiotic intervention strategies in human populations, particularly for infants at risk of intestinal dysbiosis or barrier dysfunction.

### 4.3. Microbial and Metabolic Remodeling: The Centrality of Tryptophan Metabolism

The 16S rRNA sequencing revealed that the compound probiotic did not drastically alter the overall microbial community structure until the post-weaning period (from day 70 onward), when significant inter-group differences in β-diversity emerged. This delayed effect suggests that the probiotics gradually reshape the gut ecosystem, possibly through metabolic cross-feeding and niche competition [50,51,52]. Notably, LEfSe analysis identified enrichment of beneficial bacteria, including *Clostridium butyricum* (a well-characterized butyrate producer) and *Faecalibacterium prausnitzii* (associated with anti-inflammatory effects in the gut), in probiotic-treated groups [53,54]. The persistence of all three administered strains until day 180 indicates that early-life administration promotes long-term colonization—a critical advantage over many probiotics that fail to establish stable engraftment.

The relatively modest effects of the probiotic intervention on overall gut microbial community structure (Figure 3A–F) are consistent with the view that early-life probiotic administration does not override host genetics, maternal transmission, and dietary factors as primary determinants of gut microbiota composition. Rather than globally reshaping the ecosystem, the intervention appears to exert its effects through more subtle mechanisms—namely, the enrichment of specific functional taxa (e.g., butyrate producers) and the elevation of key bioactive metabolites such as ILA. This ‘functional modulation’ perspective aligns with emerging views that probiotics may exert beneficial effects through metabolic reprogramming even in the absence of major taxonomic shifts.

Metabolomic analysis identified tryptophan metabolism as a key pathway enriched in the probiotic-treated groups, with ILA being the most consistently elevated metabolite at both days 25 and 70. This finding is particularly noteworthy given the emerging recognition of tryptophan-derived indole metabolites as central mediators of host–microbiota crosstalk. Tryptophan can be metabolized by the host along the kynurenine and serotonin pathways, or by the gut microbiota along the indole pathway. The latter yields a diverse array of AhR ligands, including ILA, indole-3-acetic acid (IAA), indole-3-aldehyde (IAId), and indole-3-propionic acid (IPA) [21,22].

Among these, ILA has drawn particular attention due to its potency as an AhR agonist and its production by select *Bifidobacterium* and *Lactobacillus* strains. Our observation that ILA was significantly elevated in the probiotic group at both days 25 and 70, whereas other indole derivatives showed less consistent increases, suggests that ILA is a signature metabolite of this particular probiotic formulation. This is consistent with studies showing that *B. infantis* grown on human milk oligosaccharides produces ILA as a predominant tryptophan metabolite, and that *L. plantarum* strains with high ILA-producing capacity activate AhR signaling to regulate intestinal homeostasis [19,55,56,57]. The fact that the three probiotic strains—*B. infantis*, *L. plantarum*, and *P. acidilactici*—may collectively contribute to ILA production through complementary metabolic activities raises the intriguing possibility of synergistic effects, though the contribution of each strain remains to be systematically dissected.

### 4.4. Mechanistic Validation: ILA Acts Through the AhR–Tight Junction Axis

The mechanistic experiments in both the DSS-induced colitis mouse model and IPEC-J2 cells provide convergent evidence that ILA exerts its barrier-protective effects. In mice, ILA administration partially alleviated DSS-induced weight loss, colon shortening, and the downregulation of tight junction proteins, while histological analysis confirmed protection against villus damage and crypt disorganization. In IPEC-J2 cells, ILA upregulated ZO-1, Occludin, and Claudin-1 expression at mRNA levels.

These findings are mechanistically consistent with a growing body of literature. ILA has been shown to protect the gut vascular barrier following intestinal ischemia injury through AhR-mediated pathways, and *L. plantarum*-derived ILA has been reported to ameliorate intestinal barrier injury by activating the AhR–Nrf2 pathway and suppressing NF-κB signaling [23,58]. The AhR, upon ligand binding, translocates to the nucleus and drives the transcription of genes encoding tight junction proteins, including ZO-1, Occludin, and Claudin-1, while also orchestrating local immune responses through the induction of IL-22 and other protective cytokines [23,59,60].

What distinguishes our study from previous reports is the identification of this mechanistic cascade in a translational large-animal model with a longitudinal design. Our findings provide evidence that ILA upregulates tight junction proteins in vivo and in vitro; however, definitive proof that the ILA–AhR axis is strictly required for the probiotic’s long-term effects in pigs (e.g., via AhR blockade or ILA depletion) awaits further investigation. Nevertheless, the cross-species conservation of this pathway supports its translational relevance.

### 4.5. Short-Chain Fatty Acids and the Multi-Faceted Nature of Probiotic Efficacy

While ILA and the AhR pathway represent a central mechanism, the beneficial effects of the compound probiotic are likely multi-faceted. We observed elevated fecal levels of acetate, propionate, and butyrate in the dual-intervention group, with butyrate showing the most pronounced increase. Butyrate serves as the primary energy source for colonocytes, promotes mucin production, and exerts anti-inflammatory effects through inhibition of histone deacetylases and modulation of regulatory T cell differentiation [54,61,62]. The enrichment of butyrate-producing bacteria such as *C. butyricum* in the probiotic-treated groups provides a plausible microbial basis for this observation.

The concurrent elevation of both ILA (an AhR agonist) and butyrate (a histone deacetylase inhibitor) in the probiotic group raises the possibility of synergistic interactions between different classes of microbial metabolites. AhR activation and HDAC inhibition may converge on common transcriptional programs involved in barrier maintenance and immune regulation, potentially amplifying the protective effects of the probiotic intervention. This hypothesis warrants further investigation, as it could inform the rational design of synbiotic formulations that combine tryptophan-metabolizing and butyrate-producing strains for enhanced efficacy.

### 4.6. Limitations and Future Directions

Several limitations of this study should be acknowledged. A notable limitation is that the three probiotic strains were administered as a fixed mixture, precluding assessment of their individual contributions to ILA production and the observed barrier-protective effects. While *B. infantis* and *L. plantarum* have both been reported to produce ILA via tryptophan transamination pathways, *P. acidilactici*—a species less extensively characterized in this context—may contribute through complementary metabolic activities or by cross-feeding interactions that enhance overall ILA yield. Systematic strain-by-strain comparative studies, ideally using mono-colonization or gnotobiotic models, will be necessary to deconvolute the relative contributions of each strain. Nevertheless, from a translational perspective, the current formulation represents a practically implementable intervention, and the additive or synergistic effects among the three strains may confer advantages over single-strain products.

Additionally, several questions remain unresolved and merit focused investigation. First, although we detected probiotic strain-specific DNA through day 180, DNA-based detection cannot distinguish live from dead cells, and the copy numbers observed in later stages may include residual DNA from non-viable bacteria. Moreover, the functional metabolic activity of persisting strains—particularly their capacity to produce ILA in situ—was not directly measured. Future studies employing viability staining, strain-specific culture, or metatranscriptomics are needed to assess the viability and functional contribution of the administered strains over time. Second, the dose–response relationship between ILA and intestinal barrier function—particularly in the context of the intact porcine gut—has not been systematically characterized; establishing the threshold and saturation concentrations for ILA-mediated AhR activation would inform the design of more precisely targeted interventions. Third, while we focused on ILA as the key metabolite, other tryptophan derivatives—such as IAId and IPA—may also contribute to AhR activation and warrant further investigation. Fourth, the study was conducted in a commercial farm setting, which inevitably introduced environmental and health-related variability. Finally, it will be important to evaluate the translational potential of this probiotic formulation in human nutritional contexts—particularly in infant populations at risk for intestinal dysbiosis or barrier dysfunction.

## 5. Conclusions

In conclusion, this study provides evidence that early-life administration of a compound probiotic comprising *Bifidobacterium longum* subsp. *infantis* BZ, *Lactobacillus plantarum* LZ, and *Pediococcus acidilactici* PZ is associated with sustained beneficial effects on intestinal barrier function and systemic growth in a translational porcine model. The three probiotic strains persisted in the gut through to adulthood (day 180), as detected by strain-specific qPCR. Multi-omics analyses revealed that the probiotic intervention enriched tryptophan metabolism and consistently elevated indole-3-lactic acid (ILA) production, with functional validation in murine and cellular models supporting a role for ILA in epithelial barrier reinforcement. Notably, administration at birth was more effective than post-weaning intervention, and two administrations (birth plus weaning) yielded superior outcomes to a single administration, highlighting the importance of early-life intervention timing. Collectively, these findings identify ILA as a key candidate metabolite and provide a mechanistic rationale for the development of probiotic-based strategies to support intestinal health during critical developmental windows. Future research should focus on optimizing strain combinations and administration protocols, as well as translating these findings to human nutritional contexts.

## Data Availability

The 16S rRNA sequencing data from porcine feces and colon contents have been deposited in the SRA database (Accession Number: PRJNA918501). The datasets used and analyzed during the current study will be available from the corresponding author, Zeqing Lu, on request.

## Supplementary Materials

The following supporting information can be downloaded at https://www.mdpi.com/article/10.3390/nu18172776/s1: Supplementary Table S1. Ingredient composition for different stages of pigs. Supplementary Table S2. Nutritional levels for different stages of pigs. Supplementary Table S3. Primers used for qRT-PCR. Supplementary Table S4. Record of piglets’ growth parameters. Supplementary Figure S1. Species discrimination analysis of LEfSe differences based on OTU levels. (A) Day 10; (B) Day 25; (C) Day 70; (D) Day 180. Supplementary Figure S2. Effects of novel compound probiotics on feces metabolites and enrichment pathways in piglets on day 10. (A) Analysis of VIP value of day 10 differential metabolites (Top 20); (B) Pathways enriched for differential metabolites on day 10 (“-” means not feeding the novel compound probiotics, “+” means feeding the novel compound probiotics, the same below). Supplementary Figure S3. Effects of novel compound probiotics on feces metabolites and enrichment pathways in piglets on day 25. (A) Analysis of VIP value of day 25 differential metabolites (Top 20); (B) Pathways enriched for differential metabolites on day 25. Supplementary Figure S4. Effects of novel compound probiotics on feces metabolites and enrichment pathways in piglets on day 70. (A) Analysis of VIP value of day 70 differential metabolites (Top 20); (B) Pathways enriched for differential metabolites on day 70.
