# Supplementary material for: Early-Life Compound Probiotic Intervention Programs Intestinal Barrier Maturation Through Indole-3-Lactic Acid in a Porcine Model
Source: Nutrients. 2026 Aug 25;18(17):2776. doi: 10.3390/nu18172776 (PMC13567698; doi:10.3390/nu18172776)
Supplement: Supplementary file 1 [file nutrients-18-02776-s001.zip › nutrients-4498445-supplementary.pdf]

Supplemental Tables

Supplementary Table S1. Ingredient composition for different stages of pigs

Feed ingredients	Day 26 - Day 110	Day 111 - Day 150	Day 151–Market
Rice，％	-	20	20
Whole-fat rice bran，％	-	5	5
Rice bran meal，％	-	3	6
Flour，％	10	5	5
First-grade corn 8.0，％	56.1	43.85	44.8
Soybean oil，％	1.5	1.5	1.5
Soybean meal，％	10	16	12
55% concentrated protein，％	2	-	-
Puffed soybeans，％	6	-	-
Corn sugar，％	1	-	-
Whey powder，％	2	-	-
Fish meal substitute，％	2	-	-
Montmorillonite，％	0.5	-	-
Enzymatic soybean meal，％	4	-	-
Finely ground stone powder，％	0.4	1.2	1.2
78.8％ 98 Lysine，％	0.5	-	-
54.6％ 70 Lysine，％	-	0.45	0.5
4％ Complete premix，％	4	4	4
Total，％	100	100	100

Supplementary Table S2. Nutritional levels for different stages of pigs

Nutritional components	Unit	Day 26 - Day 110	Day 111 - Day 150	Day 151–Market
Moisture content	%	11.95	12.31	12.25
Crude protein	%	17.37	15.43	14.29
Digestible Energy	Kcal/Kg	3239	3144	3107
Net energy	Kcal/Kg	2362	2312	2310
Crude fat	%	5	4.51	4.54
Crude fiber	%	2.27	3.52	3.63
Crude ash	%	4.49	4.84	4.8
Salt content	%	0.41	0.4	0.4
Calcium content	%	1.09	1.05	1
Phosphorus content	%	0.64	0.55	0.53
Lysine	%	1.3162	0.955	0.9055
Cu	mg/Kg	100.12	10.21	10.21
Fe	mg/Kg	0.34	61.97	61.98
Zn	mg/Kg	1535.78	41	41
Mn	mg/Kg	8.13	41.05	41.05
I	mg/Kg	1.4	3.1	3.1
Se	mg/Kg	0.07	0.23	4.7
Vitamin A	IU/Kg	12000	4200.21	4200.21
Vitamin D3	IU/Kg	2400	650.03	650.03
Vitamin E	mg/Kg	68.76	26.65	76.65
Vitamin K3	mg/Kg	7.91	1.45	1.45
Vitamin B1	mg/Kg	4.54	6.2	6.2
Vitamin B2	mg/Kg	8.15	1.4	1.4
Vitamin B6	mg/Kg	3.43	2.45	2.45
Vitamin B12	mg/Kg	1.74	-	-
Folic acid	mg/Kg	12.11	33.41	41.84
Vitamin H2	mg/Kg	0.19	2.78	3.47
Nicotinamide	mg/Kg	42.94	21.26	21.32
Calcium pantothenate	mg/Kg	12.02	8.46	8.47

Supplementary Table S3. Primers used for qRT-PCR

Gene	Primers（5'→3'）
ZO-1	F：CGTTCCGGGGAAGTTACGTG
	R：GTGGGACAAAAGTCCGGGAA
Claudin1	F：TATGACCCCTTGACCCCCAT
	R：AGAGGTTGTTTTCCGGGGAC
Occludin	F：GGTCCTCCTGGCTCAGTTG
	R：GAGTACGCTGGCTGAGAGAG
β-actin	F：GGCGGACTGTTACTGAGCTG
	R：TGCTCCAACCAACTGCTGTC
The name of probiotics	Primers（5'→3'）
Bifidobacterium longum subsp. infantis BZ	F：GAAGATGGCGAAGCCGCA
	R：CCTTGGCACGGGTCGTT
Lactobacillus plantarum LZ	F：GCGGCGTGCCTAATACATGCA
	R：TCGGACCATGCGGTCCAAGTT
Pediococcus acidilactici PZ	F：CAGGGAAAACTACGGTTGCGC
	R：CTAAAGCGTCCGCGATTGCC

Supplementary Table S4. Record of piglets' growth parameters

**Item**	**Group**			
**Lactation Stage** (Day 1 to day 26)	**Group AB**		**Group CD**	
Initial number	627		634	
Ending number	593		602	
Live rate, %	94.6		95.0	
Birth weight, kg	1.62±0.27		1.65±0.29	
Weaning weight, kg	6.50±0.78		6.85±0.86	
**Post-weaning period** (Day 26 to day 70)	**Group A**	**Group B**	**Group C**	**Group D**
Initial number	295	298	300	302
Ending number	275	285	285	292
Initial weight, kg	6.44±0.77	6.56±0.80	6.89±0.85	6.80±0.94
Ending weight, kg	19.74±0.28^{c}	19.94±0.50^{bc}	20.59±0.25^{b}	21.65±0.61^{a}
Average daily gain, g	361.98±20.82^{b}	371.63±13.96^{b}	380.57±6.99^{b}	412.68±16.93^{a}
Diarrhea rate, %	6.04±0.90^{a}	3.85±0.46^{b}	3.31±0.40^{b}	2.57±0.34^{b}

Supplemental Figures

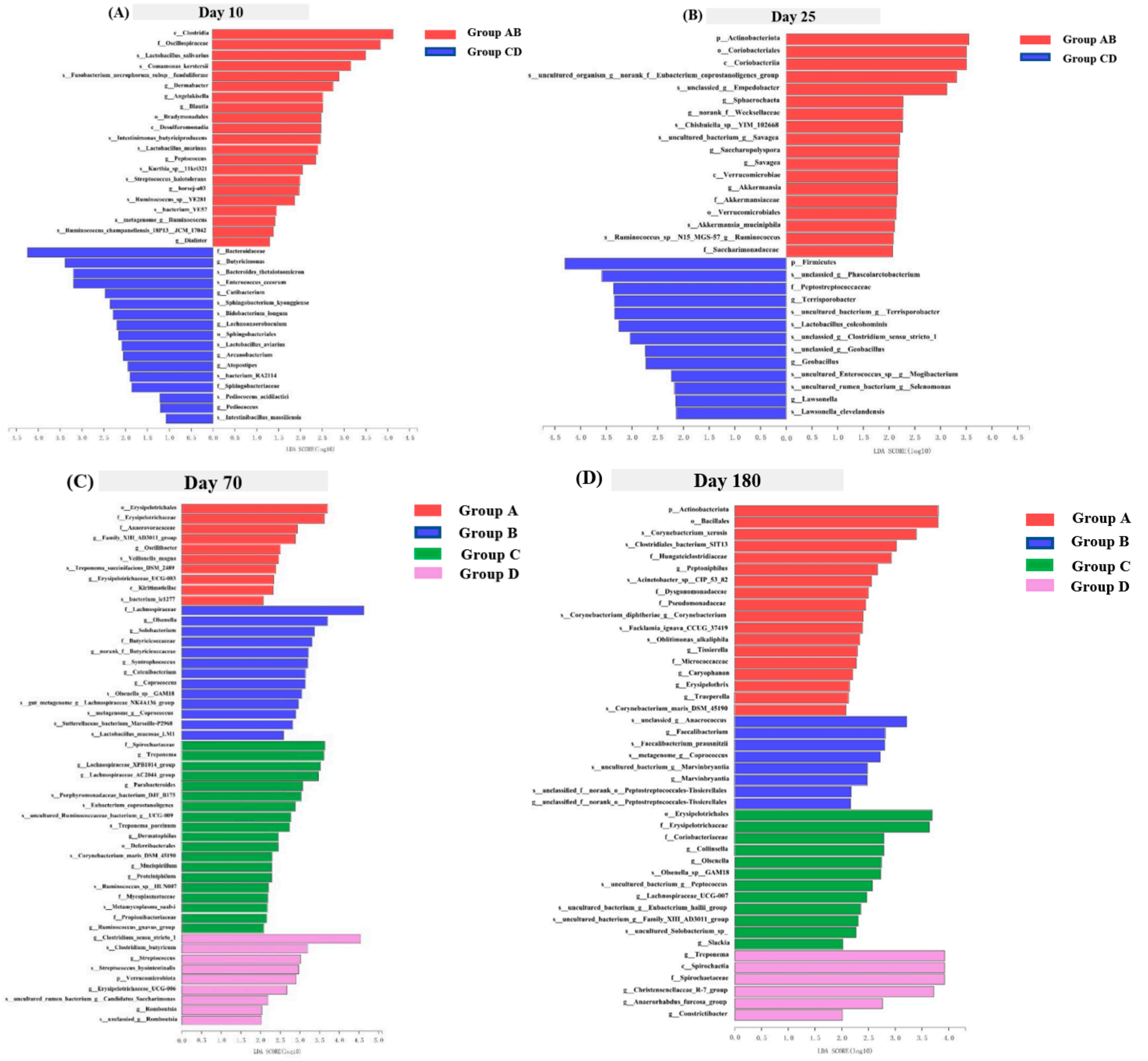

Supplementary Figure S1. Species discrimination analysis of LEfSe differences based on OTU levels. (A) Day 10; (B) Day 25; (C) Day 70; (D) Day 180.

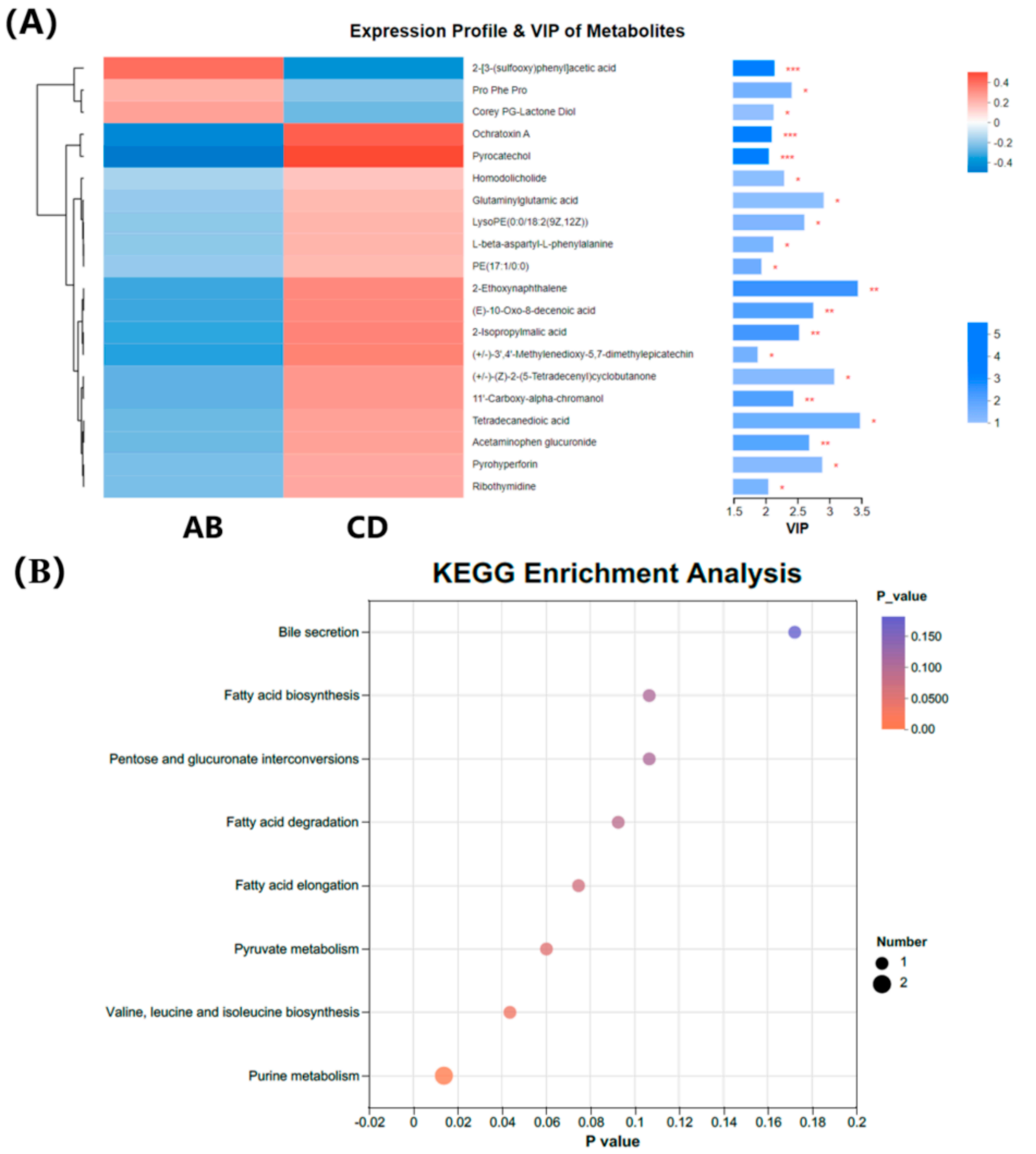

Supplementary Figure S2. Effects of novel compound probiotics on feces metabolites and enrichment pathways in piglets on day 10. (A) Analysis of VIP value of day 10 differential metabolites (Top 20); (B) Pathways enriched for differential metabolites on day 10 ("-" means not feeding the novel compound probiotics, "+" means feeding the novel compound probiotics, the same below)

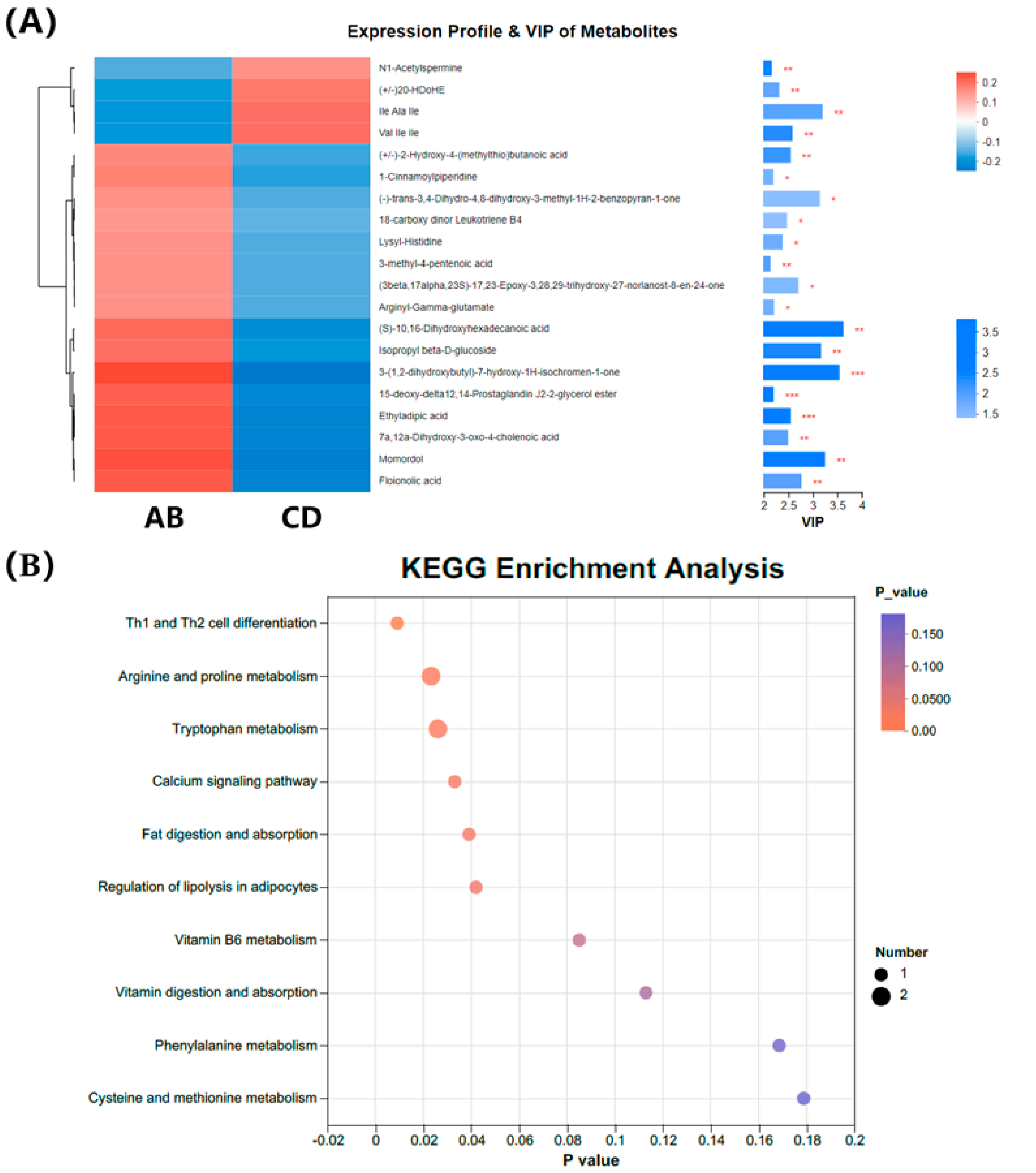

Supplementary Figure S3. Effects of novel compound probiotics on feces metabolites and enrichment pathways in piglets on day 25. (A) Analysis of VIP value of day 25 differential metabolites (Top 20); (B) Pathways enriched for differential metabolites on day 25.

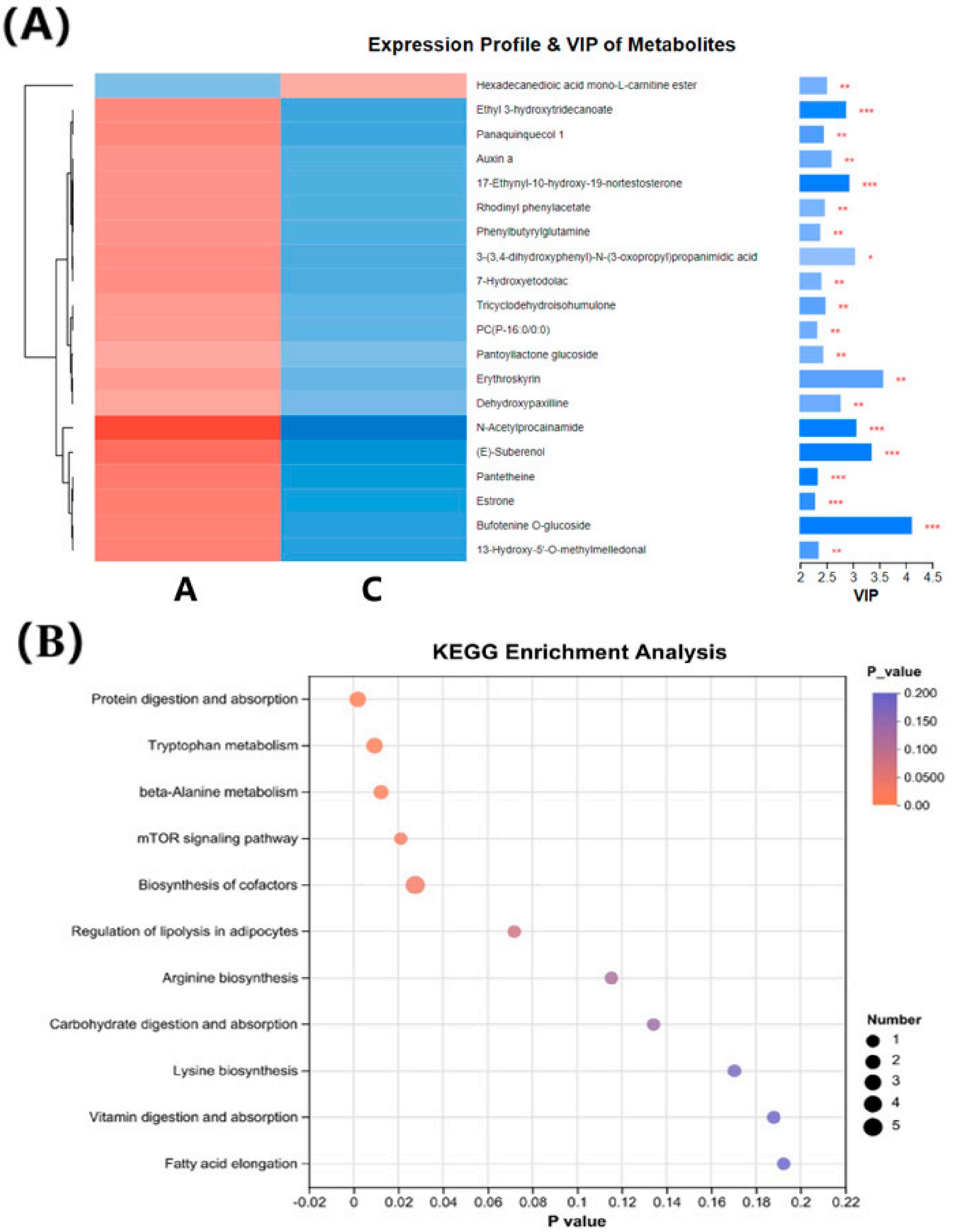

Supplementary Figure S4. Effects of novel compound probiotics on feces metabolites and enrichment pathways in piglets on day 70. (A) Analysis of VIP value of day 70 differential metabolites (Top 20); (B) Pathways enriched for differential metabolites on day 70.
